# Don’t Pack a Pest: Leishmania Guyanensis in Southwest Michigan

**DOI:** 10.7759/cureus.80956

**Published:** 2025-03-21

**Authors:** Samantha M Campbell, Alan Furlan, Bruna Dellatorre Diniz, Francisco F Costa Filho, Harry Boamah

**Affiliations:** 1 Internal Medicine, Western Michigan University, Kalamazoo, USA; 2 Infectious Disease, Western Michigan University, Kalamazoo, USA

**Keywords:** cutaneous leishmaniasis, new world leishmaniasis, rash cutaneous lesions, treatment of leishmaniasis, zoonotic infectious disease

## Abstract

Leishmaniasis is a protozoal infection that is typically transmitted through the saliva of a female Phlebotomine sandfly and can result in devastating mucocutaneous infections if not identified and treated quickly. Here we present a case of cutaneous *Leishmania guyanensis* in a 49-year-old woman who had recently gone on a trip to Costa Rica and was unaware of any bites she may have sustained while being there. The patient initially presented with a facial rash and was seen by both her primary care physician and dermatology before receiving a referral to infectious disease. After a prolonged work-up, during which she was given antibiotics and steroids, skin biopsy was able to confirm the diagnosis of *Leishmania guyanensis*, and she was started on a four-week course of miltefosine with complete resolution of her rash.

## Introduction

Leishmania is a protozoan that has become one of the leading causes of subcutaneous and mucosal infections throughout the Central and South Americas, Africa, and Asia. Its primary mode of transmission is through bites from the Phlebotomine sandfly, which mainly targets dogs and humans. While both female and male sandflies feed on sugar-rich liquids, it is the female fly only that can pierce through skin and blood-feed with the purpose of producing eggs. The saliva of the sandfly aids in the feeding process, and it is within the salvia that the Leishmania protozoa live. To date, more than 20 different species of Leishmania have been identified using PCR [1].

The type of infection can be categorized into two main subtypes based on geographic location: New World (Mexico, Central America, and South America) and Old World (southern Europe, tropical regions of Africa, the Middle East, and Asia). The primary difference between the two is that New World Leishmaniasis produces mucocutaneous infection along with the cutaneous and visceral disease that are seen in Old World infection [2].

Currently, most mucocutanenous Leishmaniasis infections are caused by the species braziliensis; however, other species such as guyanensis, amazonesis, and panamensis have been identified as causing these infections as well [3]. Infection typically presents as a single ulcerated lesion that can spontaneously heal. It has been noted that in northern Brazil it can typically present as multiple lesions [2]. The infection may also be accompanied by lymphadenopathy. Oral mucosal involvement can be seen more often in male patients compared to female patients. There is also a high incidence of perforation of the nasal septum when the nasal mucosa is involved [4]. Identification can be done in a few ways. The least invasive option would be IgM and IgG antibodies, and it can also be identified through skin biopsy. Amphotericin B was historically the first-line treatment; however, due to its extensive side effect profile, other medications, such as pentamidine and phosphocholines, are now preferred.

## Case presentation

A 49-year-old female with a past medical history of hypothyroidism, abnormal uterine bleeding (status/post hysterectomy), and depression presented to the Infectious Disease clinic for evaluation of a rash on her left cheek. The rash initially started as a small "bump," which she assumed to be a pimple, developing after she had returned home from an eight-day work trip to Costa Rica. She denied any insect or tick bites on any part of her body. As time progressed, the area became more erythematous, spreading to the size of a dime, and was raised with an irregular texture. She works as a program director at a youth camp, and the trip to Costa Rica was one of the scheduled activities. During the trip, she visited local villages, went white-water rafting, and visited volcanos. She primarily stayed in hotels during her trip. None of the students who had accompanied her developed any illness or any rashes upon arrival back to the United States. She denied recent viral illnesses or having this type of rash in the past. No other rashes or lesions were noted on other parts of her body.

Two months after initial development of the lesion, she went to her primary care provider, who referred her to dermatology. At that time, she had developed a painful rash overlying the lesion, which had a burning, itching quality, but no blistering was seen. She was initially prescribed clindamycin for three days; however, no improvement was seen and thus she was placed on doxycycline for one week. She was also started on prednisone, with a 16-day taper. She reported that after the initial regimen of antibiotics and steroids, while her lesion improved, the rash did not, and thus she was started once more on a prednisone taper and was given doxycycline for 30 days. This longer course of treatment still did not improve her rash, and she noted that the lesions would return towards the end of her steroid taper. It was then recommended that she pursue treatment at an Infectious Disease clinic.

On initial evaluation at the Infectious Disease clinic, her rash had progressed and was involved the entire left upper check, without involvement of the eyes, mucosal membranes of nares, or the oral cavity. No one in her household had similar rashes, and she had not had any recent changes in medications or personal care products. With the lack of response to medication, it was decided that a skin biopsy should be performed. Doxycycline was replaced with trimethoprim/sulfamethoxazole while awaiting official biopsy report.

**Figure 1:**
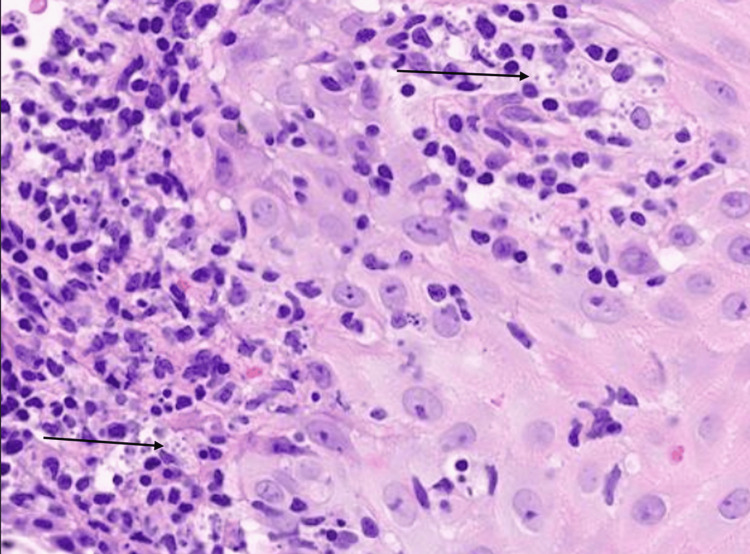
High-powered (100x magnification) H&E slide showing brisk and diffuse inflammatory infiltrate of lymphocytes, histiocytes, and scattered neutrophils. Organisms are seen within histiocytes. This is most consistent with cutaneous leishmaniasis. Arrows pointing to small organisms within histiocytes.

Initial biopsy reported a cutaneous leishmaniasis (Figure 1), and a second biopsy was obtained with DNA extraction and molecular testing to define the subspecies. The patient was placed on miltefosine (50mg three times daily) for 28 days with pending results. After two weeks, her facial rash had improved, and by the end of the 28-day course, the rash had completely resolved and she was left with a small residual scar from the biopsy. Final PCR testing confirmed the species as being *Leishmania guyanensis*.

## Discussion

Considering epidemiology, it is uncommon to see Leishmaniasis in the U.S. However, with the rise in travel, tropical diseases are increasingly appearing in the U.S. [4]. This case highlights the importance of a thorough travel history of a patient, as it could give important clues to infectious etiologies.

With the patient’s location of recent travel, there should be a heightened suspicion for diseases endemic to the country. The improvement of her lesion with doxycycline and prednisone possibly caused a delay in diagnosis, with a premature assumption that her infection was bacterial in nature. The prednisone; however, likely suppressed her inflammatory response enough to mimic partial improvement, until repeat treatment had failed to improve the rash. As Leishmaniasis incubation ranges from weeks to months [2], it is also possible that the progression of her rash was due to the incubation time coming to an end.

This case emphasizes the importance of conducting skin biopsies and PCR testing for rashes that do not respond to conventional treatment.  Leishmaniasis can develop into a severe disease that significantly affects a person's appearance, and so early identification and treatment is important to ensure no mucosal involvement and need for debridement or removal of bone/tissue. Failure to diagnose can lead to complete destruction of nasal structures, including the nasal septum. A case-series done in 2019 revealed that loss of the nasal septum occurred in 25% of patients who did not have a timely diagnosis [5]. PCR testing can be increasingly important, as some species are more aggressive than others, particularly Braziliensis, Panamensis, and Guyanesis [3]. 

As Leishmaniasis is seen more often in the U.S., more treatment options are coming on the market. At one point, Amphotericin-B was predominantly used as treatment, particularly in pregnant women [6]. With its extensive side-effect profile, however, there have been studies to find medications that are better tolerated. Pentamidine can be used, as it interferes with polyamine synthesis, therefore RNA polymerase activity. It enters the protozoan cells and prevents synthesis of proteins, nucleic acids, phospholipids, and folate. It’s only FDA approved indication at this time is for Pneumocystis Jiroveci pneumonia in high-risk HIV patients as it is quite toxic to not only protozoa and bacteria but also to humans [7]. Miltefosine, while a FDA approved treatment for Leishmaniasis, it is not currently approved for the species seen in this case. It disrupts Ca2+ homeostasis and activates the Ca2+ plasma membrane channel within Leishmaniasis species. This causes an increase in Ca2+ within the mitochondrial membrane, disrupting electrochemical membrane potential and therefore death of the parasite. These actions appear to be parasite specific, which limits the side effect profile of this medication, making it the safest option for treatment currently [8,9]. It is imperative to involve surgical intervention if an eschar or abscess is present. As these infections can cause open ulcers, any super-imposed bacterial infection should be treated appropriately to limit complications.

## Conclusions

While still relatively rare in developed countries, there is an increasing number of cases of mucocutaneous Leishmaniasis seen in travelers coming from endemic countries. Due to its epidemiology, it can often be misdiagnosed at first contact, leading to delayed treatment. This can result in devastating complications if delayed for too long. This patient had a classic presentation of the disease, emphasizing the importance of biopsy with PCR (particularly as there are three species known to cause mucocutaneous Leishmaniasis), good history taking including travel and lodging accommodations, and the prompt involvement of infectious disease.
